# Trans–right atrial access to the left ventricle for catheter ablation of ventricular tachycardia in a patient with double left-sided mechanical valves: First case report from Latin America

**DOI:** 10.1016/j.hroo.2025.08.028

**Published:** 2025-08-21

**Authors:** Luis Quiñiñir, Pablo Salazar, Pasquale Santangeli

**Affiliations:** 1 Internal Medicine Department, Universidad de La Frontera School of Medicine, Temuco, Chile; 2 Electrophysiology Section, Cardiovascular Division, Hospital Dr. Hernan Henriquez Aravena, Temuco, Chile; 3 Electrophysiology Section, Cardiovascular Division, Cleveland Clinic, Cleveland, Ohio

**Keywords:** Ventricular tachycardia, Catheter ablation, Double mechanical valves, Aortic and mitral mechanical valves, Trans–right atrial access

**Key Findings**

▪ Transatrial access via the inferoseptal process of the left ventricle provides a feasible and safe route for endocardial ventricular tachycardia ablation in patients with mechanical aortic and mitral valves.
▪ Intracardiac echocardiography is essential for precise puncture guidance, minimizing risks to adjacent structures and improving procedural safety.
▪ To our knowledge, this is the first reported case in Latin America, demonstrating global reproducibility and highlighting the need for further innovation and training to meet the needs of underserved populations with a high prevalence of rheumatic heart disease.

## Introduction

Patients with combined mechanical aortic and mitral valve prostheses pose a unique challenge for catheter ablation of arrhythmias arising from the left ventricle (LV), as the traditional access routes are rendered impossible. Therefore, unconventional strategies, such as epicardial access, transcoronary venous catheterization, surgical transapical access, or intentional septal defects, have been explored. Santangeli et al^1^ developed a percutaneous technique from the right atrium (RA) through the inferoseptal process (ISP), mirroring a Gerbode defect and allowing catheter access for endocardial ventricular tachycardia (VT) ablation. Reproducibility of this approach on a global scale is essential to increase patient access to life-altering VT ablation procedures. We present the first description of this technique reported in Latin America.

## Case description

A 61-year-old man with interstitial lung disease and advanced heart failure, with an LV ejection fraction of 30% secondary to rheumatic heart disease (RHD), and with mechanical prosthetic valves in both the aortic and mitral positions, as well as cardiac resynchronization therapy with defibrillator, presented with an electrical storm and multiple appropriate implantable cardioverter-defibrillator (ICD) shocks refractory to antiarrhythmic treatment. After multidisciplinary evaluation, catheter ablation was recommended. However, the usual LV endocardial access routes were contraindicated by his double prosthetic mechanical valves. Therefore, trans-RA LV access via the ISP was proposed. Written informed consent was obtained from the patient for the procedure and case report publication. This report adhered to the revised 2013 Helsinki Declaration guidelines.

### Procedural approach

The procedure was performed under general anesthesia. Vascular access was obtained under ultrasound guidance. Warfarin was uninterrupted, and a heparin infusion was administered and titrated to achieve a goal of activated clotting time >300 s during the entire procedure. Intracardiac echocardiography (ICE) was used to visualize the cardiac structures and to guide the entire procedure.

1. *Targeting the puncture site:* A long deflectable sheath (Vizigo, Biosense Webster, Inc) was advanced into the RA. Under ICE guidance, the sheath was deflected toward the inferior-septal region of the RA, adjacent to the ISP-LV. The optimal site was identified near the inferior aspect of the coronary sinus ostium. Firm contact of the sheath tip on the RA side of the ISP-LV was confirmed (Figure 1).

**Figure 1:**
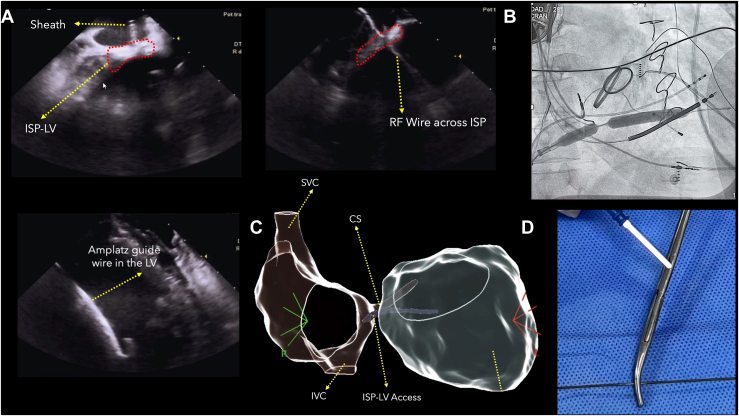
**A:** Intracardiac echocardiography (ICE) showing the deflectable sheath positioned in the right atrium (RA) and directed toward the inferoseptal process of the left ventricle (ISP-LV) (left) as well as a successful radiofrequency (RF) wire crossing into the LV (right). **B:** Fluoroscopic right anterior oblique projection demonstrating inflation of a 9-mm noncompliant balloon at the RA-to-LV puncture site to dilate the tract. **C:** CARTO 3-dimensional electroanatomic map showing RA anatomy and the ISP-LV access site, near the inferior third of the coronary sinus (CS) ostium. **D:** Custom-modified RF wire with an exposed distal segment (metallic), designed to conduct energy precisely across the septum under ICE and fluoroscopic guidance. IVC = inferior vena cava; SVC = superior vena cava.
2. *RA-LV puncture:* Specifically designed radiofrequency wires are not commercially available in our country; instead, a 0.035-in guidewire (Amplatz, Cook Medical) was introduced through the sheath with the stiff tip at the end. The surface metal of a small portion at the proximal end of the wire was exposed to allow contact with electrocautery. Then, the wire was advanced while applying energy (≈25 W for ∼15 seconds), perforating the ISP-LV. Successful LV access was visualized on ICE and fluoroscopy.
3. *Wire exchange:* Upon gaining access, a 4-F hydrophilic microcatheter (Terumo GlideCath) was passed over the radiofrequency wire into the LV. Through this microcatheter, the radiofrequency wire was exchanged for a stiff 0.035-in Amplatz Super Stiff guidewire. The Amplatz wire was positioned securely in the LV apex, providing a rail for dilation and sheath advancement.
4. *Tract dilation and sheath insertion:* Over the Amplatz wire, a noncompliant angioplasty balloon (9 mm diameter × 40 mm length) was advanced and 2 serial dilations were performed. Then, the Vizigo sheath was advanced over the balloon.
5. *Mapping and ablation:* High-density electroanatomic mapping was performed using a mapping catheter (PentaRay, Biosense Webster). Programmed electrical stimulation from the right ventricular apex did not induce any VT under general anesthesia. Therefore, a substrate-based ablation approach was pursued. Voltage mapping in sinus rhythm revealed an extensive inferolateral LV scar, spanning the basal to mid-inferolateral wall. Within the scar, late potentials and fractionated electrograms were identified. Catheter ablation was performed using an irrigated contact force catheter (ThermoCool SmartTouch SF, Biosense Webster), and multiple radiofrequency ablation lesions (45–50 W, temperature-controlled) targeting these abnormal potentials were performed. Programmed stimulation was then repeated from the right ventricular apex, and no sustained VT could be induced postablation. The total procedure time was approximately 4.5 hours, with about 50 minutes required to obtain LV access.

The patient had an uneventful recovery. No new arrhythmias or conduction disturbances occurred. In particular, he maintained atrioventricular conduction (intact atrioventricular node function)—an important consideration given the septal vicinity of the puncture, but in line with prior series where no heart block was observed. His ICD was reprogrammed, and no further appropriate shocks or VT episodes were noted during the index hospitalization. The patient was discharged on day 5 with optimized heart failure therapy and continuation of anticoagulation. At 1-month follow-up, the patient remains free of VT recurrence and ICD therapies, and no evident ventricular septal defect was visible on transthoracic echocardiography upon follow-up.

## Discussion

VT ablation in patients with double left-sided mechanical prosthetic valves represents a major challenge. Attempts at conventional LV access via retrograde aortic or transseptal approaches have been associated with serious complications, including acute valve dysfunction and death.^2^ Nevertheless, recurrent VT often arises from LV substrates, demanding innovative solutions.

We describe a successful application of a percutaneous trans-RA approach to the ISP-LV, originally detailed in 2020 by Santangeli et al^1^ and later validated in a multicenter registry.^3^ The technique has demonstrated a favorable safety profile. In the 4-patient single-center experience, no access-related complications occurred. The multicenter registry of 18 patients reported one self-limited perimitral hematoma managed conservatively.^3^ Importantly, no new atrioventricular block was observed in patients with baseline intact conduction in either study.

To our knowledge, this report is the first to describe the application of this technique in Latin America, where its relevance may be particularly high given the regional burden of RHD. In 2015, countries with endemic RHD, predominantly low- and middle-income nations including much of Latin America, had an age-standardized prevalence of 444 cases per 100,000, compared with just 3.4 cases per 100,000, in nonendemic, high-income countries. Subregions such as the Caribbean, Andean, and tropical Latin America report rates ranging from 100 to 300 cases per 100,000, underscoring a disproportionately high prevalence of valvular sequelae.^4^ Since surgical valve replacement remains a cornerstone of care for RHD in these settings, this increases the likelihood of encountering patients with double mechanical valves requiring future VT ablation.

As such, trans-RA to LV access may be of utility in Latin American countries, where the intersection of structural heart disease and arrhythmia burden remains clinically significant. Wider dissemination of this technique, particularly in public health systems, could expand access to lifesaving VT ablation in anatomically complex patients. Additionally, with the increasing use of percutaneous structural interventions such as transcatheter aortic valves and mitral clip devices, where preserving prosthesis integrity is critical, this approach may have utility in those populations as well.

Future development of dedicated puncture tools and steerable sheaths tailored for this approach may further enhance procedural efficiency and safety. Likewise, broader training in ICE imaging and refinement of catheter navigation technologies may facilitate adoption in centers currently lacking this expertise.

In conclusion, the RA-to-LV ISP puncture technique provides a viable, reproducible, and safe alternative for endocardial LV access in patients with double mechanical valves. The effective implementation of this approach in Latin America demonstrates its potential for global expansion and underscores the importance of continued innovation and professional development to address the needs of underserved populations and varied clinical settings.
